# Hydroclorate Ammonia in Neuralgia

**Published:** 1855-05

**Authors:** 

**Affiliations:** The Bengal Medical Service


### HYDROCHLORATE OF AMMONIA INTERNALLY IN NEURALGIA.

By Dr. Ebden, of the Bengal Medical Service.

Carrying out Dr. Watson's recommendation, Dr. Ebden has tried this remedy "in a great many instances and cases, and with, almost invariably, satisfactory results." He writes:

"In facial neuralgia, tic-doloureaux, nervous head-ache, tooth-ache, clavus-hystericus, and in affections of this neuralgic kind generally, and not excepting sciatica, and even in one case of neuralgic dysmenorrhœa, I have often given it, and have been convinced, after a full trial of its merits, that it is decidedly a very valuable and powerful remedy for the relief of neuralgic pain generally.

"I usually prescribed from twenty-five to thirty five grains of the salt in an ounce of mint water, or camphor mixture every twenty minutes, for three doses, giving, if required, a saline aperient with the first dose. The second dose is usually sufficient for the relief of the immediate pain; but I have observed that where it has been necessary to repeat and continue the doses, the patient has, in many instances, afterwards, enjoyed a comparative immunity from the recurrence of pain; and therefore have I, in some cases, been led to continue the exhibition of the muriate systematically at six or eight hours' intervals for some days. From memoranda of many satisfactory cases, I am induced here to select the particulars of two, in which the good effects were great and marked."

1. In June, 1850, at Simla, a lady of somewhat delicate frame, æt. 35, suffered very severely from an attack of facial neuralgia, an affection to which she was very subject. She had travelled all over Europe, and had, in many large cities, consulted professional savans on this disease, to which she was so great a martyr. No advice had benefitted her, "no doctors' stuff yet had ever given her any relief." After some persuasion, but with no hope on her part of success, she was induced to try the muriate in full doses. While in actual agony, she took the first thirty grs. with marked relief, in ten minutes' time; the second dose *quite* removed all pain. She has never since had any return of her old enemy, for now she wards off every threatening attack, with a dose of "the ammonia muriate" solution, with which she is always now provided.

2. A clergyman who had suffered terribly from "nervous headaches" coming on at all times, but having apparently no other disorder of his general health, had consulted many medical men, and taken many remedies. Early in 1851, he tried the ammonia with the immediate relief to the present attack, and with very great *alleviation* of many subsequent headaches. He, too, managed to ward off very many attacks by taking 30 grs. of the muriate whenever the pain threatened; and he was rendered, after some few days' treatment, very much less liable to them, than he had previously been for many years.—*Indian Annals Medical Science.*

